# Effects of Eating Fresh Lean Pork on Cardiometabolic Health Parameters

**DOI:** 10.3390/nu4070711

**Published:** 2012-07-05

**Authors:** Karen J. Murphy, Rebecca L. Thomson, Alison M. Coates, Jonathan D. Buckley, Peter R. C. Howe

**Affiliations:** Nutritional Physiology Research Centre, University of South Australia, GPO Box 2471, Adelaide, South Australia 5000, Australia; Email: rebecca.thomson@unisa.edu.au (R.L.T.); alison.coates@unisa.edu.au (A.M.C.); jonathan.buckley@unisa.edu.au (J.D.B.); peter.howe@unisa.edu.au (P.R.C.H.)

**Keywords:** pork, cardiometabolic health, body composition, BMI

## Abstract

High protein meat-based diets are commonly promoted for weight loss, supposedly by increasing satiety and energy expenditure. Pork is a good source of protein however little information on the metabolic effects of pork consumption exists. This pilot study aimed to examine whether regular consumption of fresh lean pork could improve body composition and cardiovascular risk factors in a 6 month parallel intervention trial. 164 overweight adults (mean BMI 32) were randomly assigned to incorporate up to 1 kg pork/week by substituting for other foods or maintain their habitual diet (control). Plasma levels of lipids, glucose and insulin, BMI, waist/hip circumference, blood pressure, heart rate and arterial compliance were measured at baseline and 3 and 6 months. Body composition was determined using dual energy X-ray absorptiometry. A total of 144 volunteers completed and volunteers in the pork group increased their intake 10 fold by substituting pork for mainly beef and chicken. After 3 months, there were significant (*p* ≤ 0.01) reductions in weight, BMI, waist circumference, % body fat, fat mass and abdominal fat in the pork group relative to controls, which persisted for 6 months. There was no change in lean mass, indicating that the reduction in weight was due to loss of fat mass. There were no significant effects on other metabolic parameters. Regular consumption of lean fresh pork may improve body composition.

## 1. Introduction

The majority of dietary intervention trials investigating effects of high protein diets on health outcomes have generally used beef as the high protein source while pork tends to be thought of less in terms of its nutritional benefit, but in fact lean pork has a similar nutrient composition to lean beef. Most dietary guidelines recommend daily consumption of lean meat to deliver key nutrients such as protein, thiamine, niacin, vitamin B12 and zinc [1]. Previous research has been conducted on other meats such as beef as part of a high protein energy restricted diet designed for weight loss [2,3,4,5,6]. It is thought that high meat protein diets may enhance weight loss by increasing satiety, leading to a reduced energy intake, while at the same time increasing thermogenesis which then blunts the normal fall in energy expenditure generally seen in weight loss [2]. 

Intervention studies have shown that high protein diets, approximately 30% energy from protein [4,5,6], containing lean cuts of meat (mainly beef) can elicit a number of improvements in cardiovascular disease (CVD) risk factors, including reduced low density lipoprotein (LDL) [7], improved glucose control and insulin sensitivity, reduced risk of type II diabetes, reduced blood pressure (BP), increased satiety [5], reduced body weight and improved weight control [3,8,9]. Parker *et al*. [5] found that consuming a energy restricted high protein diet for 3 months reduced total cholesterol, triglycerides (TAG) and LDL. McMillan-Price *et al*. [10] demonstrated that consumption of an energy restricted medium protein (25% of energy from protein), reduced fat, high fiber diet with either high or low glycemic index for 3 months resulted in mean reductions of ~6% in body weight, ~6 cm in waist circumference and 4 kg in fat mass as measured by dual energy X-ray absorptiometry (DEXA).

Little research has focussed on the potential cardiometabolic health benefits of consuming lean pork. Yet, given that pork has a similar nutrition profile to other meats, regular consumption of fresh lean pork may be expected to deliver similar cardiovascular and metabolic health benefits. Moreover, there is some evidence that pork may be more satiating than other meats [11]. Therefore the aim of the present pilot study was to evaluate the impact of regular consumption of fresh lean pork on body composition and risk factors for diabetes and CVD.

## 2. Experimental Section

### 2.1. Subjects

Free-living overweight/obese, non-smoking men and women, aged 18–65 years who were low pork consumers (ate less than 1 pork meal a week) were recruited through local media advertisements to participate in a 6 month, randomized, controlled, parallel intervention trial. Subjects were excluded if they reported one of the following: diagnosed diabetes or CVD; history of myocardial infarction or stroke; peripheral vascular disease; BP > 160/100 mmHg; liver or renal disease; anti-inflammatory, antihypertensive or hypocholesterolemic drug therapy that was not stable in the previous 3 months; already eating >100 g fresh pork per week; inability to consume pork as required. Eligible volunteers were stratified according to gender, BMI and age and randomly allocated to one of two groups. Randomization was by minimization [12]. This study was conducted according to the guidelines laid down in the Declaration of Helsinki and all procedures involving human subjects were approved by the Human Research Ethics Committee (19/12/2007) at the University of South Australia, Adelaide, Australia. Written informed consent was obtained from all subjects. The trial was registered on the Australia New Zealand Clinical Trials Register (ACTRN12608000190303, 11/4/2008). 

#### 2.1.1. Study Design

**Figure 1:**
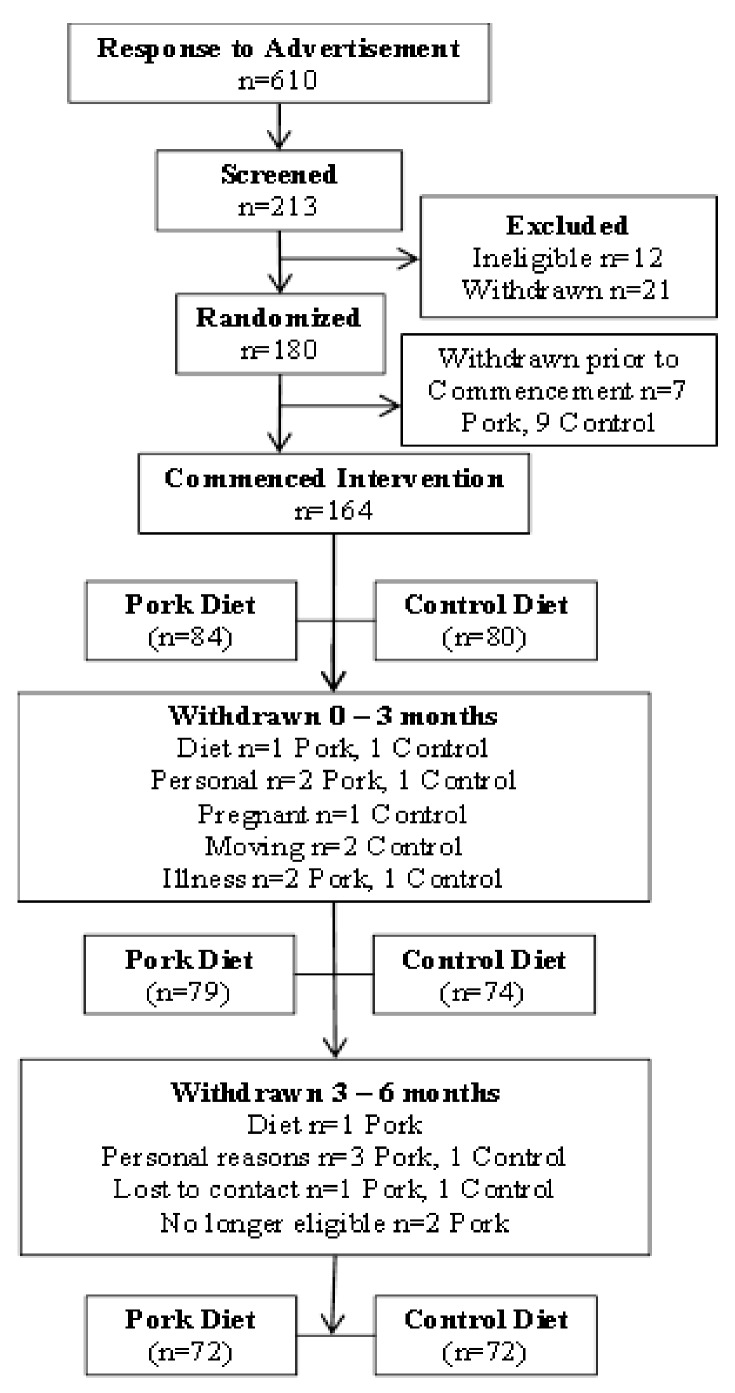
Consort diagram.

A parallel design intervention trial was conducted to determine whether there were any differences in body composition (primary measure was percent body fat) following pork consumption for 3 or 6 months compared with habitual diet consumption. Hence only volunteers for whom data was collected at each time point were included in the final analyses. Of a total of 213 volunteers who were screened for eligibility, 184 were randomized to either consume fresh lean pork for 6 months or remain on their habitual diet (control, <100 g fresh pork per week) for 6 months and 164 commenced the study (Figure 1). Subjects attended the clinic on two consecutive days at baseline and after 3 and 6 months of intervention and the following assessments were made at each time point unless stated otherwise. Weight was recorded to calculate BMI (kg/m^2^; height was measured at baseline only). Waist and hip circumference, BP, arterial compliance, heart rate, dietary intake and physical activity levels were measured using food frequency questionnaires and physical activity diaries, respectively, and a fasted blood sample was taken on each of the two consecutive clinic days. 

#### 2.1.2. Dietary Intervention

Subjects in the pork group (*n* = 84) were seen fortnightly to monitor body weight, discuss any issues arising in the intervention and collect a selection of frozen pork comprising 150 g servings of lean steak, stir fry, diced, mince and sausages. They were instructed to incorporate 7 servings (men) or 5 servings (women) of pork per week into their habitual diet for 6 months and to keep a weekly log of pork consumption. Recipe books were provided to volunteers in the pork group with low fat and salt recipes for each pork cut to help maintain compliance. It was completely up to each volunteers how they would incorporate pork into their regular diet. Subjects in the control group (*n* = 80) were asked to maintain their normal diet for 6 months and were followed up regularly with phone calls to see how they were progressing and to discuss any issues arising in the 6 month period.

#### 2.1.3. Dietary Intake

Dietary intakes of total energy and macronutrients were estimated using a 74-item food frequency questionnaire from the Cancer Council of Victoria, Australia (FFQ) which requests information relating to food choices, frequency, portion size, quantity and consumption rate of different food and beverage items. The FFQ has been validated by Xingying and colleagues [13] in human clinical trials reflecting dietary intake in the last 12 months. The FFQ was adjusted to incorporate the macronutrient profile of the study pork (Table 1). Meat was purchased from a single supplier to minimize variations in butchering and preparation of lean cuts. The energy, moisture, ash and nutrient composition of the pork was analyzed by George Weston Technologies (NSW, Australia).

**Table 1:** Nutritional composition of lean fresh pork per 100g *.

	Diced	Stir-fry	Steak	Mince	Sausage
Energy (kJ)	580	425	770	611	790
Fat (g)	6.8	1.1	10.8	7.0	13.5
Saturated Fat (g)	2.8	0.4	4.6	2.7	5.2
Protein (g)	19.3	21.4	21.8	20.7	14.3
Total carbohydrate (g)	<1.0	1.2	<1.0	<1.0	2.8
Moisture (g)	73.3	74.8	67.2	71.9	66.6
Sodium (mg)	333	346	204	182	933

* Energy, moisture, ash and nutrient composition of the pork was analyzed by George Weston Technologies (NSW, Australia).

#### 2.1.4. Physical Activity

Subjects recorded a diary of all physical activity conducted in a 24 h period over 3 days (2 weekdays and 1 weekend day) [14]. Energy expenditure (kcal) was then calculated for every 15 min period in a 24 h day according to 9 categories of different types of activity (e.g., sleeping, playing sports, gardening *etc*.) and multiplied by the appropriate physical activity level factor for the reported intensity of exercise. This was multiplied by body weight, then averaged for 3 days. 

#### 2.1.5. Body Composition

Each subject’s height and weight were recorded to calculate body mass index (BMI; in kg/m^2^). Waist and hip measurements were taken using a metric tape measure according to international guidelines, as described by Norton and Olds [15]. Subjects had their percentage of body fat, fat mass, abdominal fat and lean mass assessed using DEXA (Lunar Prodigy, General Electric, Madison, WI, USA). Repeated assessments made on consecutive days in 11 overweight or obese subjects gave the following standard errors for measures of body composition: 0.87% for percentage body fat, 0.53 kg for fat mass and 1.05 kg for lean mass.

#### 2.1.6. Blood Pressure and Arterial Compliance

Systolic and diastolic BP, heart rate and indices of compliance in proximal (large capacitance) arteries (C1) and distal (small resistance) arteries (C2) were measured as described elsewhere [16].

#### 2.1.7. Laboratory Methods-Sample Collection

Fasted venous blood was drawn into Vacutainer tubes containing EDTA or sodium citrate (Vacuette, Greiner bio-one, Austria) at baseline and 3 and 6 months. Plasma was separated by centrifuging the blood at 1800× *g* for 10 min at 4 °C and stored at −80 °C until analysis. 

#### 2.1.8. Blood Lipids

Plasma total cholesterol (TC), triglycerides and HDL cholesterol were determined enzymatically on the Konelab analyser (Thermo Electron Corporation, Louisville, CO, USA) with reagents from ThermoFisher Scientific (Melbourne, Australia). Low density lipoprotein (LDL) cholesterol was calculated using the Friedewald formula [17]. Triglycerides were measured from two fasted samples on consecutive days to gain an average value. Samples were measured in a single assay to minimise inter-assay variation. 

#### 2.1.9. Plasma Glucose and Insulin

Plasma glucose concentrations were measured on a Konelab analyser (Thermo Electron Corporation, Louisville, CO, USA) using a commercial enzymatic kit (ThermoFisher Scientific, Melbourne, Australia). Plasma insulin concentrations were determined using a human insulin specific radioimmunoassay (Abacus-ALS, Millipore, MO, USA).

#### 2.1.10. Statistical Analysis

Based on previous determinations of the variance in the primary outcome measure (change of % body fat from baseline to 6 months), we estimated that a total of 140 subjects would give 90% power to observe a significant difference in % body fat of 2% at an alpha level of 0.05. Data of subjects who completed the trial were analyzed using Repeated Measures ANOVA with Bonferroni *post hoc* analyses to identify differences between means where significant main effects were seen. Analysis focused on changes in variables from baseline to 6 months and intermediate analysis from baseline to 3 months, between groups and within groups using SPSS 18 (SPSS, Chicago, IL, USA). Data are presented as means ± SEM (standard error of mean). Significance was set at *p* < 0.05 unless otherwise stated. 

## 3. Results

Of the 164 subjects who commenced the intervention, 3 withdrew due to diet related issues while 17 withdrew due to personal reasons, were no longer eligible or lost to contact. Thus 144 subjects (*n* = 72, pork diet and *n* = 72, habitual diet) completed the full 6-month intervention period. There were no statistical differences in baseline characteristics of volunteers who withdrew or completed the study. Mean age and height of subjects in the pork and control groups was 48 ± 12 years and 1.7 ± 0.1 m, respectively. There were no significant differences in age, height, weight, BMI, waist or hip circumference, body composition, BP, heart rate, arterial compliance, plasma lipids, glucose, insulin, dietary intake or total energy expenditure between the two groups at baseline. 

**Figure 2:**
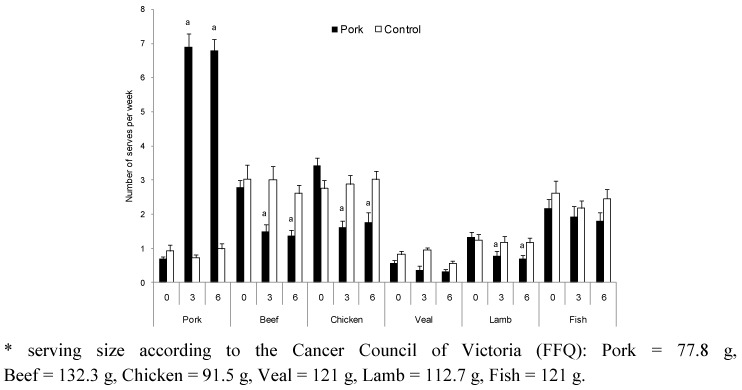
Average meat and fish consumption (mean ± SEM, servings/week) from the Cancer Council of Victoria Food Frequency Questionnaire for pork (*n* = 71) and control (*n* = 72) groups at baseline and 3 and 6 months. ^a^ Significant change from 0 month (*p* < 0.001).

Consumption of provided pork was calculated from the daily pork consumption logs. Men in the pork group were provided with 1050 g of fresh lean pork per week and consumed 946 g per week (135 g/day) on average. Women in the pork group were provided with 750 g of fresh lean pork per week and consumed 682 g (97 g/day) per week on average. According to baseline intakes of pork as estimated from the FFQ, the pork and control groups were consuming less than 1 serving of pork per week, prior to commencement of the study. The pork group increased their intake from 0.7 servings per week to 6.9 and 6.8 servings per week at 3 and 6 months of the study respectively, whereas intakes of beef, chicken and lamb decreased by approximately 50% at 3 and 6 months (*p* < 0.001; Figure 2). There were no significant changes in consumption of veal or fish (Figure 2). Total meat intake (sum of pork, chicken, beef, veal and fish) for the pork group was 201, 205 and 199 g/day at 0, 3 and 6 months, respectively, and 210, 194 and 198 g/day at 0, 3 and 6 months, respectively, for the control group. 

There was no difference in energy intake (kJ) (Table 2) or macronutrients (total fat, protein or carbohydrate) in either group over time. This indicates that volunteers in the pork group were substituting meats in their diet without impacting on total energy or total protein intake. There was no difference in total energy expenditure (kJ/day) according to the physical activity diaries, indicating that subjects in both groups did not change their physical activity levels or energy expenditure during the intervention. A number of volunteers failed to return their physical activity diaries explaining the fewer number of subjects. 

**Table 2:** Average values for energy, macronutrients and total energy expenditure of volunteers in the pork and control groups at baseline and 3 and 6 months.

	Pork								Control					
	*n*	0 month		3 months		6 months		*n*	0 month		3 months		6 months	
		Mean	SEM	Mean	SEM	Mean	SEM		Mean	SEM	Mean	SEM	Mean	SEM
Energy (kJ)	*71*	8690	341	8151	413	8188	488	*72*	8955	457	8397	390	8284	378
Protein (%en)	*71*	20	0.4	20	0.4	20	0.3	*72*	20	0.5	20	0.4	21	0.5
Fat (%en)	*71*	35	0.6	33	0.6	33	0.7	*72*	35	0.7	35	0.7	35	0.7
SFA (g)	*71*	32	1.5	31	2.0	31	2.2	*72*	33	2.2	32	1.9	30	1.8
MUFA (g)	*71*	30	1.3	29	1.6	29	2.1	*72*	31	2.0	29	1.6	29	1.6
PUFA (g)	*71*	12	0.7	11	0.6	11	0.8	*72*	13	0.8	12	0.7	12	0.6
CHO (%en)	*71*	40	0.8	41	0.9	41	0.8	*72*	40	0.8	39	0.8	38	0.9
Alcohol (%en)	*71*	5	0.8	5	0.8	6	0.8	*72*	6	0.9	6	0.9	6	0.9
TEE (kJ/day)	*61*	16296	462	16761	508	16511	464	*56*	16632	111	16611	431	16760	477

Abbreviations: kJ, kilojoules; %en, percent of energy; CHO, carbohydrate; TEE, total energy expenditure; No significant differences were reported for any variable.

All volunteers had normal values (<135/85 mmHg) for systolic and diastolic BP at baseline (Table 3) but levels of total cholesterol, LDL cholesterol and plasma glucose were borderline high [18]. There were no changes over time in any cardiovascular (CV) or metabolic parameters measured in either the pork or control groups.

**Figure 3:**
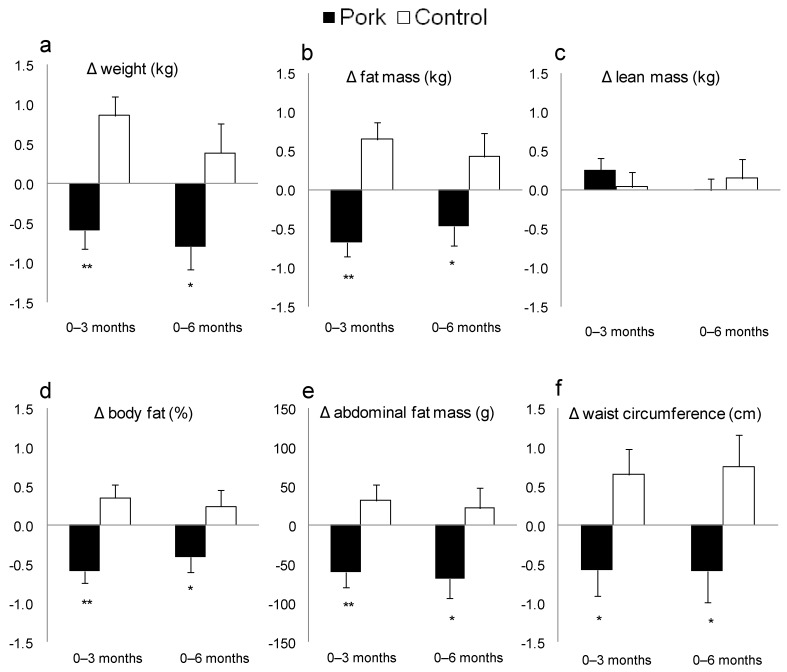
Change in (**a**) weight (kg); (**b**) fat mass (kg); (**c**) lean mass (kg); (**d**) body fat (%); (**e**) abdominal fat mass (g); and (**f**) waist circumference (cm) for pork and control groups 3 and 6 months. Values are means ± SEM. * Significantly different from change in control group (*p* < 0.05), ** Significantly different from change in control group (*p* ≤ 0.001).

During the first 3 months of intervention, those who remained on their customary diet significantly increased their weight, BMI and fat mass (Table 3). In contrast, subjects in the pork group significantly reduced these measures of adiposity during this period. Compared with the control group, the pork group significantly improved their weight (pork: −0.7 ± 0.2 kg at 3 months and −0.8 ± 0.3 kg at 6 months *vs*. control: 0.8 ± 0.2 kg at 3 months and 0.4 ± 0.4 kg at 6 months), BMI, waist circumference and body composition including % body fat, fat mass and abdominal fat (time × treatment effect: *p* < 0.01 in all cases, Table 3). These relative improvements in measures of adiposity were still evident after 6 months of intervention (Figure 3). However, there was no change in lean mass (Table 3 and Figure 3), which indicates that the reduction in weight was due to loss of fat mass.

**Table 3:** Average values for blood vessel function, blood lipids, glucose and insulin and body composition of volunteers in the pork and control groups at baseline and 3 and 6 months.

	Pork								Control					
	*n*	0 month		3 months		6 months		*n*	0 month		3 months		6 months	
		Mean	SEM	Mean	SEM	Mean	SEM		Mean	SEM	Mean	SEM	Mean	SEM
SBP (mmHg)	*72*	126	1.6	123	1.4	124	1.4	*72*	127	1.5	125	1.4	126	1.5
DBP (mmHg)	*72*	73	1.1	71	1.0	71	1.1	*72*	72	1.0	70	1.0	70	1.1
Heart rate (bpm)	*72*	62	1.1	61	1.1	60	1.1	*72*	61	1.0	61	1.0	60	1.1
Large artery EI (mL/mmHg × 10)	*72*	17.1	0.5	16.4	0.5	16.8	0.5	*71*	16.7	0.6	16.3	0.5	16.3	0.5
Small artery EI (mL/mmHg × 10)	*72*	7.9	0.4	7.9	0.4	8.1	0.4	*71*	8.3	0.4	8.8	0.5	8.6	0.5
TC (mmol/L)	*72*	5.6	0.1	5.6	0.1	5.5	0.1	*71*	5.8	0.1	5.7	0.1	5.6	0.1
LDL (mmol/L)	*71*	3.7	0.1	3.6	0.1	3.6	0.1	*69*	3.7	0.1	3.7	0.1	3.6	0.1
HDL (mmol/L)	*72*	1.3	0.04	1.3	0.03	1.3	0.03	*71*	1.4	0.03	1.4	0.03	1.3	0.03
TAG (mmol/L)	*71*	1.5	0.1	1.4	0.1	1.3	0.1	*69*	1.4	0.1	1.4	0.1	1.3	0.1
Gluc (mmol/L)	*71*	5.9	0.1	5.8	0.1	5.8	0.1	*70*	5.9	0.1	5.9	0.1	5.9	0.1
Insulin (µU/mL)	*68*	20.6	1.1	19.6	1.0	18.5	0.9	*67*	19.4	0.8	18.6	0.9	18.8	0.8
Weight (kg) ^2^	*72*	91.4	2.1	90.7 ^3^	2.1	90.6	2.1	*72*	92.8	2.0	93.6 ^4^	2.0	93.2	2.0
BMI (kg/m^2^) ^2^	*72*	31.8	0.6	31.5 ^3^	0.6	31.5	0.7	*72*	31.9	0.5	32.3 ^3^	0.5	32.1	0.5
WC (cm) ^2^	*72*	101.3	1.8	100.7	1.8	100.7	1.8	*72*	101.3	1.6	102.0	1.6	102.1	1.6
HC (cm)	*72*	112.6	1.4	112.1	1.4	112.2	1.4	*72*	111.8	1.1	111.9	1.1	111.9	1.1
% fat ^2^	*72*	42.5	1.1	41.9 ^4^	1.1	42.1	1.1	*71*	40.9	1.0	41.3	1.0	41.1	1.0
Fat mass (kg) ^ 2^	*72*	37.2	1.3	36.5 ^4^	1.4	36.7	1.4	*71*	36.2	1.1	36.8 ^3^	1.2	36.6	1.1
Abdo fat (g) ^ 2^	*72*	3025	145	2960 ^4^	144	2957 ^3^	148	*71*	3006	128	3037	131	3028	131
Lean mass (kg)	*72*	49.9	1.4	50.1	1.4	49.9	1.4	*71*	52.6	1.5	52.6	1.5	52.7	1.5

Abbreviations: SBP, systolic blood pressure; DBP, diastolic blood pressure; mmHg, millimeters of mercury; bpm, beats per minute; EI, elasticity index; mL/mm Hg × 10, milliliters per millimeters of mercury; TC, total cholesterol; Gluc, glucose; WC, waist circumference; HC, hip circumference; abdo, abdominal; ^2^ Significant treatment × time effect (*p* < 0.01), Significantly different from 0 month ^3^ (*p* < 0.05), ^4^ (*p*≤ 0.001).

## 4. Discussion

Previous studies have focussed on relationships between the consumption of lean red meat and increased satiety and weight loss [3,5]. Most of this research has utilised hypocaloric, high protein diets specifically designed for weight loss, with little research to date investigating the cardiometabolic health benefits of eating fresh lean pork. The aim of the present study was to determine the effect of regular consumption of fresh lean pork on body composition and CV risk factors over a 6 month period, with no energy restriction and without change in habitual physical activity patterns. 

The present study found that in those consuming pork, body composition was improved compared to controls, as shown by modest reductions in weight, fat mass, % body fat, abdominal fat and waist circumference, without loss of lean mass or any adverse effects on CV risk factors measured. These improvements in body composition were achieved without changes in total meat or protein intake. This study is the first to our knowledge to show improvements in body composition with regular pork consumption. 

Pork is less popular than beef and chicken in Australian diets, as reflected by consumption levels in the National Nutrition Survey [19] and the present study wherein subjects habitually ate less than 1 serving of pork compared with 2.5–3 servings of both beef and chicken per week. The lesser consumption of pork probably reflects a common misconception that it is an unhealthy meat rich in saturated fat; the link between saturated fat and CVD is likely to influence consumers’ choice of meat. We were able to demonstrate in the present study that adult volunteers could readily increase their intakes of lean fresh pork cuts to nearly 7 servings per week in place of beef and chicken for 6 months without affecting either their total meat intake or the CV risk factors assessed. This observation has important implications for pork producers and consumers alike. 

The means by which the pork diet achieved improvements in body composition compared with the habitual diet is unclear, although a subtle difference in energy balance cannot be ruled out. We estimate that a change as small as 400 kJ/day could account for the observed changes in body composition. Even though there was no significant change in energy intake in the current study, the FFQ, while validated in clinical trials, might not have been sufficiently sensitive to detect such a subtle change in energy intake. Similar limitations apply to our ability to estimate energy expenditure. Interestingly, Mikkelsen and colleagues [20] showed greater 24 h energy expenditure (thermogenesis) following a pork diet than a soy diet or high carbohydrate diet. Fat levels were matched in all 3 diets, protein was matched on the pork and soy diets and energy intake did not change during the intervention. It appears that the thermogenic effect of protein depends on the type of protein and it may be that the type and amount of amino acids present in pork protein favor increased protein synthesis and turnover rates which in turn increase thermogenesis and energy expenditure leading to less fat deposition. However, we are unable to say if the improvements in the present study were specific to pork or whether consumption of other high protein meat diets would have had the same effect. 

Based on past experiences in our centre, volunteers appear to participate in our dietary intervention trials to learn more about their health such as information on their blood lipid and glucose profiles, blood pressure, body fat and dietary intake therefore are very compliant with the study protocol. However we do acknowledge that the difference in contact with the control group during the trial is a potential confounder. Moreover Wadden *et al*. [21] demonstrated maintenance of weight loss in participants from the Look AHEAD study in those participants who attended more treatment sessions. As described earlier, the pork group met with the investigators fortnightly whereas the control group was contacted regularly by phone to see how they were progressing. Thus patterns of diet and exercise in the latter group may have fluctuated more between visits, reducing the reliability of assessments of energy intake and expenditure. 

There has been much discussion about the association between meat consumption and development of coronary heart disease, stroke and diabetes, most likely due to concern over the saturated fat content of meats and its effect on CV risk factors such as blood cholesterol levels. However, relationships between meat consumption and cardiometabolic health parameters are not well defined. A recent systematic review and meta-analysis of the evidence for relationships between unprocessed fresh meat from beef, hamburgers, lamb, pork or game and processed meat (any meat preserved by smoking, curing, salting, or addition of chemical preservatives such as bacon, salami, sausages, hot dogs or processed deli or luncheon meats) found that the intake of unprocessed (fresh) meat was not associated with coronary heart disease or diabetes mellitus, whereas processed meat intake was associated with 42% higher risk of coronary heart disease and 19% higher risk of diabetes mellitus [22]. This study demonstrates the need for greater understanding of the potential cardiometabolic health benefits of fresh lean meat and recognition in dietary recommendations. 

The present study found no change in a selection of CV risk factors following regular consumption of fresh lean pork for 6 months. These results are in agreement with Coates *et al*. [23] who showed that consumption of 1 kg of fresh pork per week for 12 weeks had no adverse effect on blood lipids. In the present study improvements in risk factors were not expected as the intervention was not intended to be a hypocaloric or high protein diet. 

## 5. Conclusions

In summary, this pilot study demonstrated that regular inclusion of lean fresh pork in the diet in place of other meats may improve body composition without adversely affecting risk factors for diabetes and CV disease. Improvements in body composition were achieved without energy restriction or apparent changes in physical activity levels, total meat or protein intakes. However, the change in body composition may reflect a subtle difference in energy balance. Further research is warranted to investigate the effect of different meat sources on energy balance and body composition using more sensitive technology. The present observations suggest that the perception of pork as a less nutritious meat should be reconsidered.
